# Supplementary material for: COVID-19 in Japan, January–March 2020: insights from the first three months of the epidemic
Source: BMC Infect Dis. 2022 May 25;22:493. doi: 10.1186/s12879-022-07469-1 (PMC9130991; doi:10.1186/s12879-022-07469-1)
Supplement: Supplementary file 1 — Additional file 1: Fig. S1. Proportion of the population (yellow) of Japan and confirmed COVID-19 cases (green) by sex and 10-year age bands. Fig. S2. Proportion of known contacts by contact type. Fig. S3. Time between symptom onset and case report for before (top panel) and after (bottom panel) the estimated peak (19 March 2020). Grey bars show the observed delay, the blue and orange bars show the best-fit gamma probability density function for the (blue) naïve delay and (orange) the delay corrected for epidemic growth and decay. Fig. S4. Change in contact exposure types (known and unknown) over time from 14 January to 31 March 2020. Fig. S5. Daily number of PCR tests conducted nationally from 5 Feb to 31 March 2020 as reported by the Ministry of Health Japan [1]. Fig. S6. Daily incidence of confirmed COVID-19 cases by date of symptom onset and the estimated time-varying reproduction number (Rt) estimated from incidence data truncated using the empirical mean onset-to-reporting delay: overall (pink) and accounting for imported cases (black). The solid line shows the median and the shaded area the 95% credible interval. Table S1. Summary of national and regional COVID-19 interventions up to 31 March 2020. Table S2. Age-specific transmission probability (95% CI, 3 d.p) matrix for all provinces in the first wave of the epidemic in Japan (N=1276). Table S3. Summary of p-values of the Kolmogorov-Smirnov statistic to assess whether samples of time from symptom onset to first occurrence of a specific symptom from different age groups are drawn from the same distribution. [file 12879_2022_7469_MOESM1_ESM.pdf]

Additional Material 1

Results

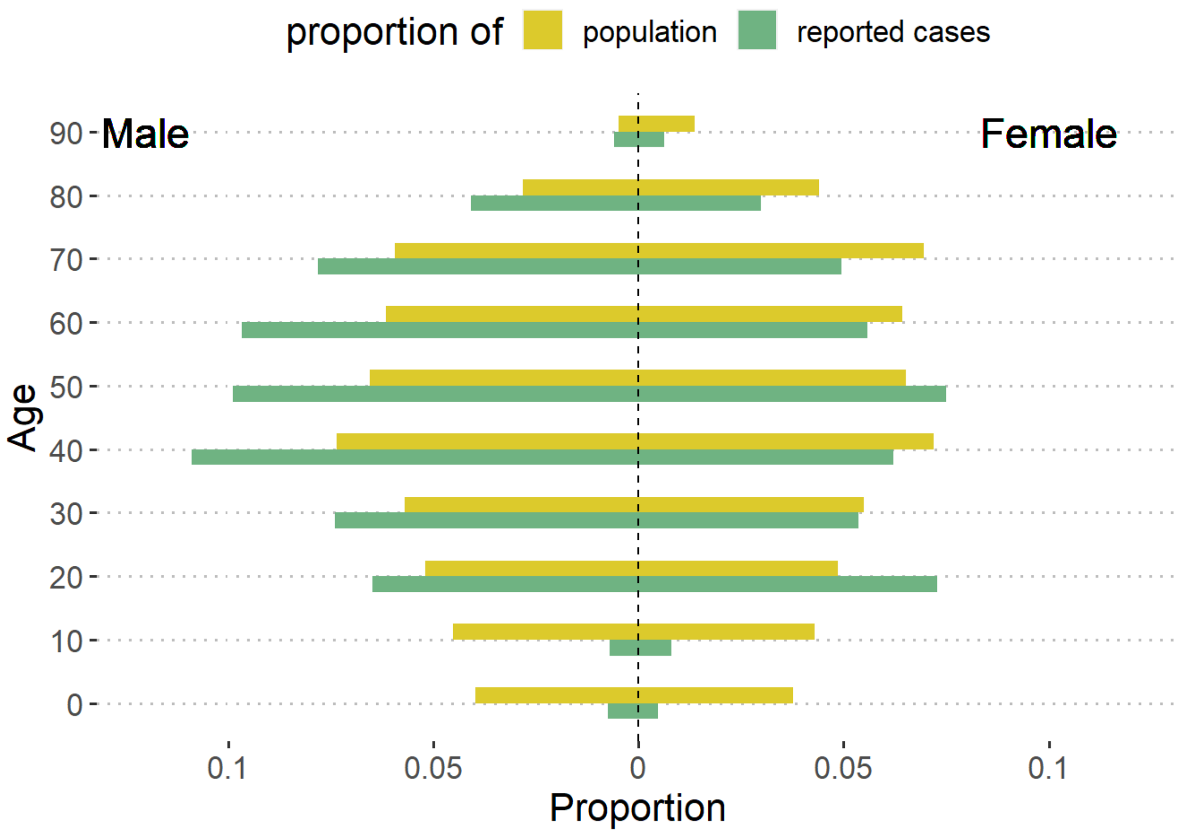

Supplementary Figure 1: Proportion of the population (yellow) of Japan and confirmed COVID-19 cases (green) by sex and 10-year age bands.

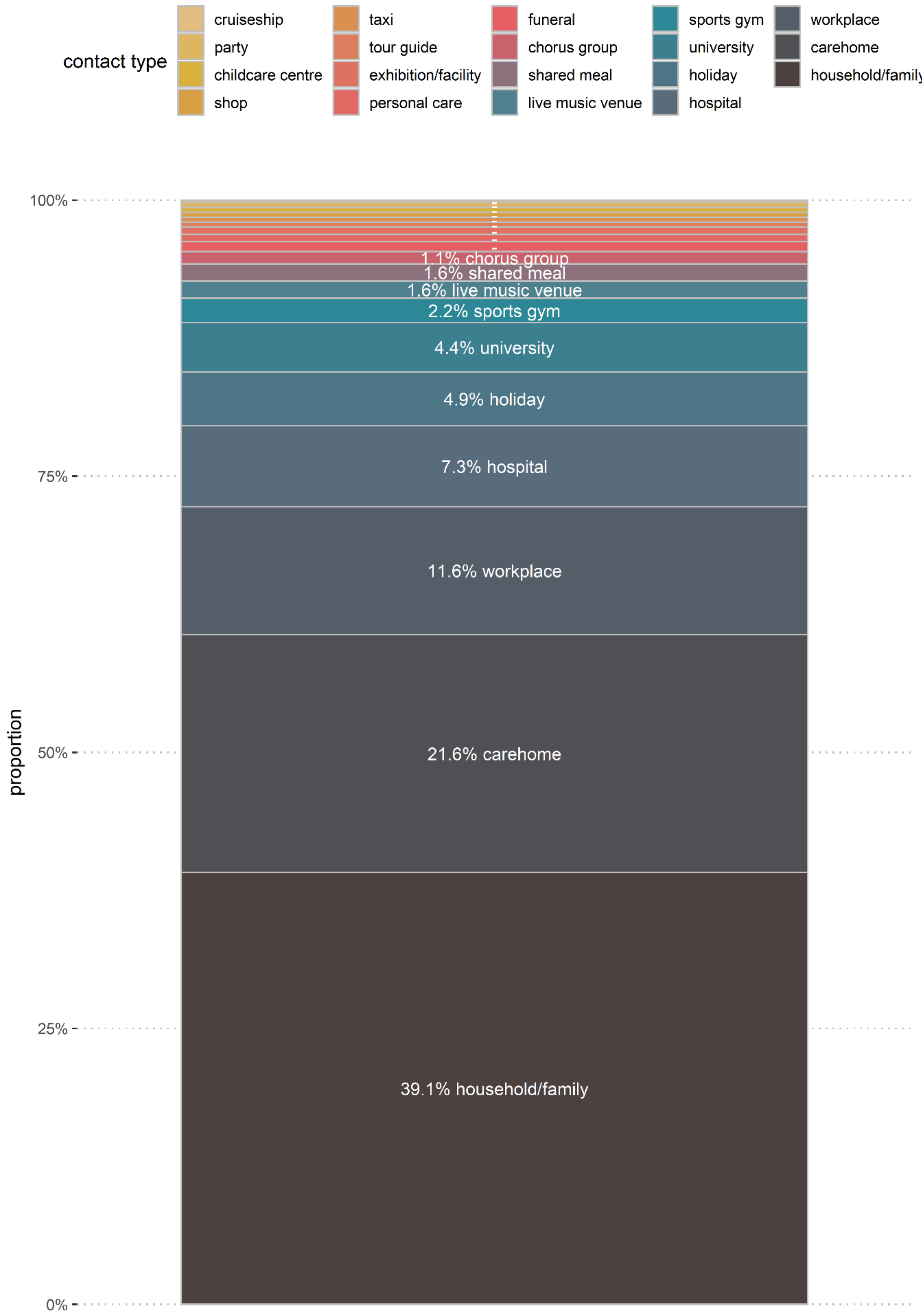

Supplementary Figure 2: Proportion of known contacts by contact type.

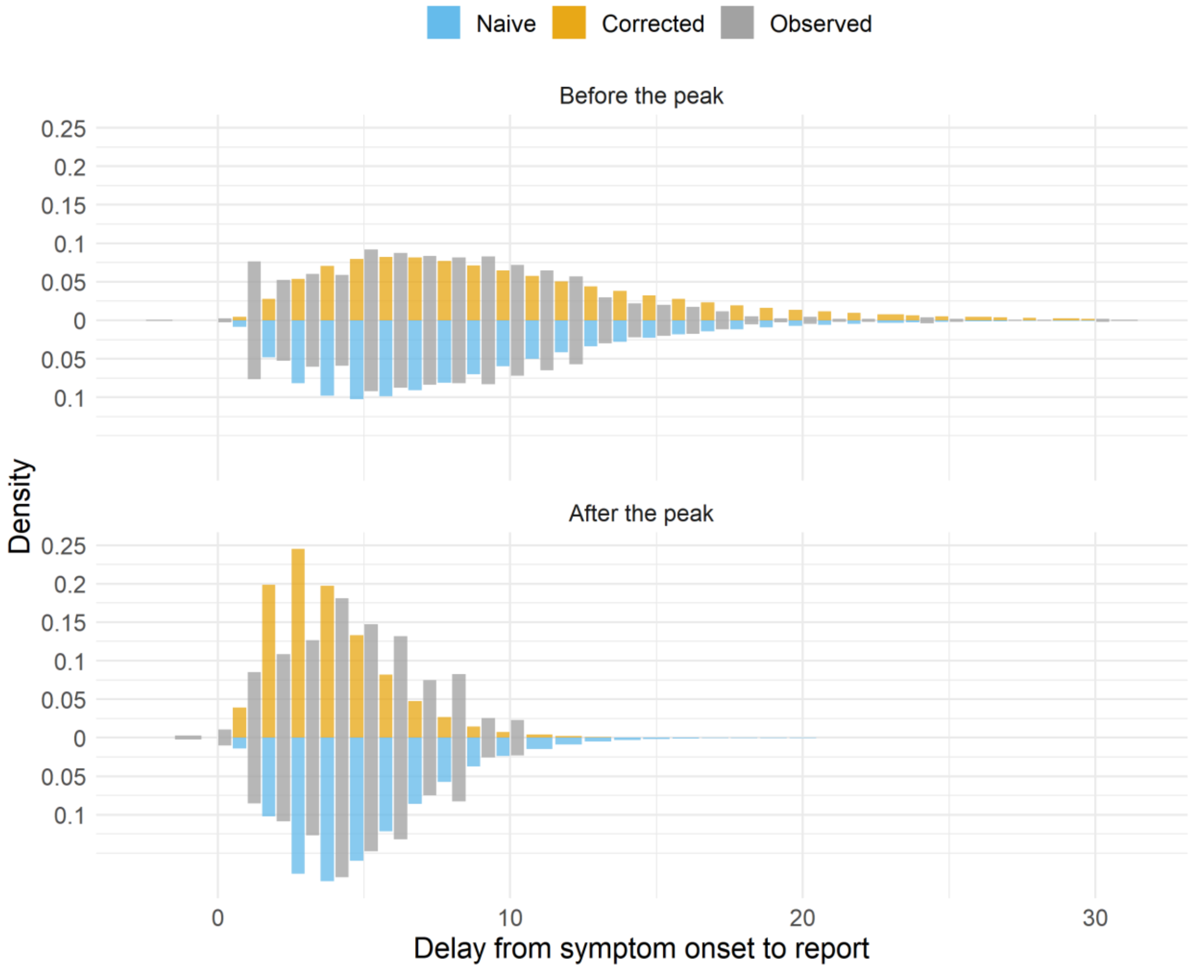

Supplementary Figure 3: Time between symptom onset and case report for before (top panel) and after (bottom panel) the estimated peak (19 March 2020). Grey bars show the observed delay, the blue and orange bars show the best-fit gamma probability density function for the (blue) naïve delay and (orange) the delay corrected for epidemic growth and decay.

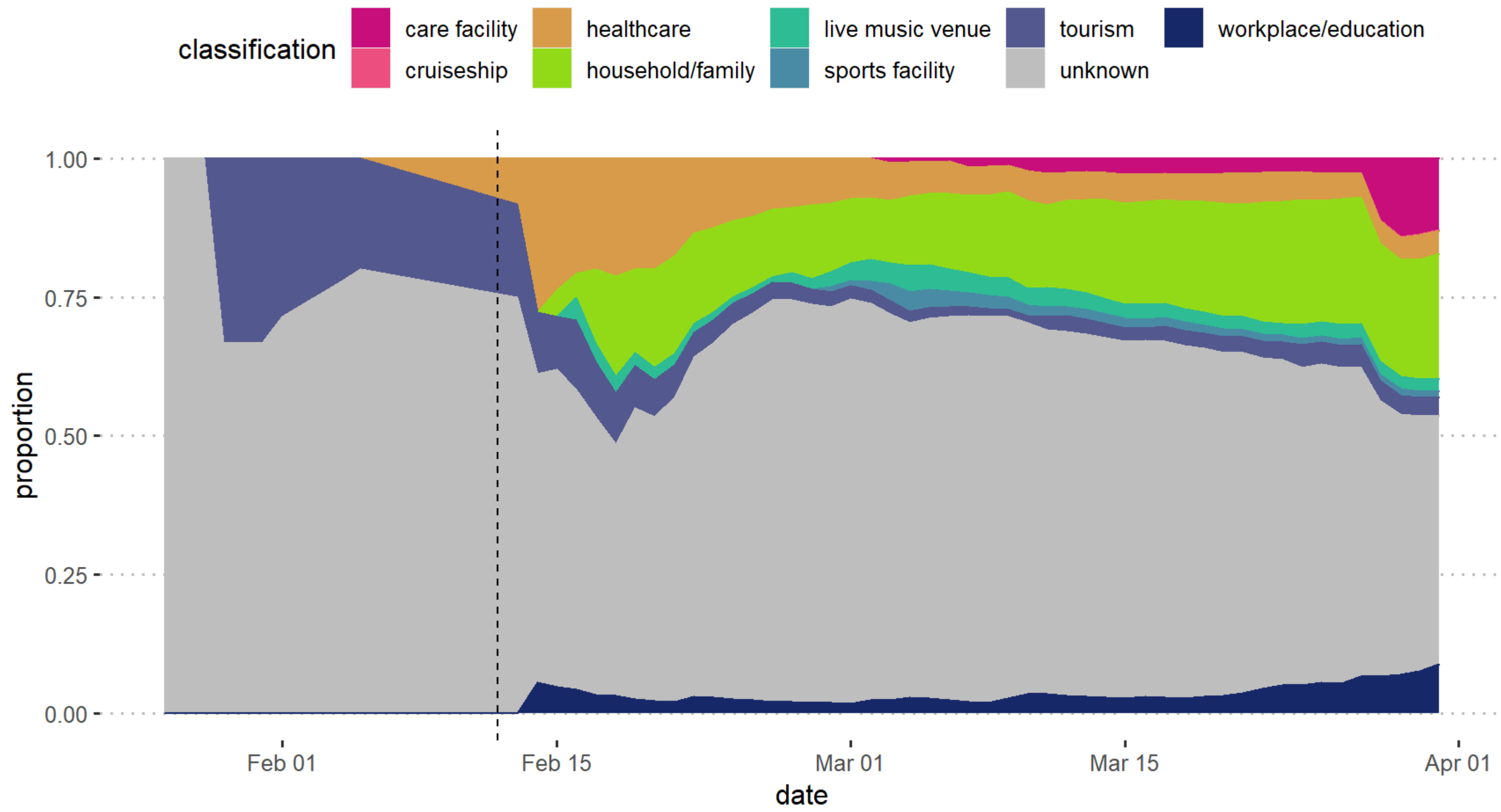

Supplementary Figure 4: Change in contact exposure types (known and unknown) over time from 14 January to 31 March 2020.

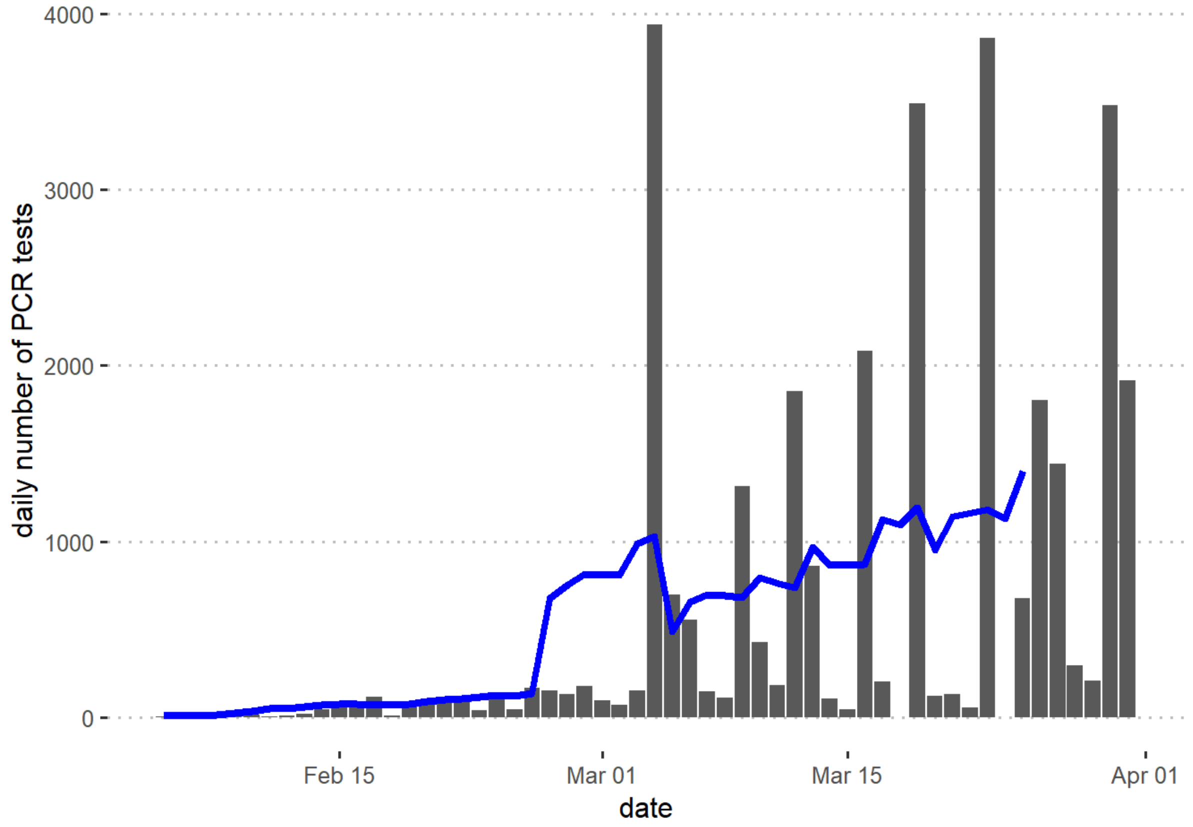

Supplementary Figure 5: Daily number of PCR tests conducted nationally from 5 Feb to 31 March 2020 as reported by the Ministry of Health Japan [1].

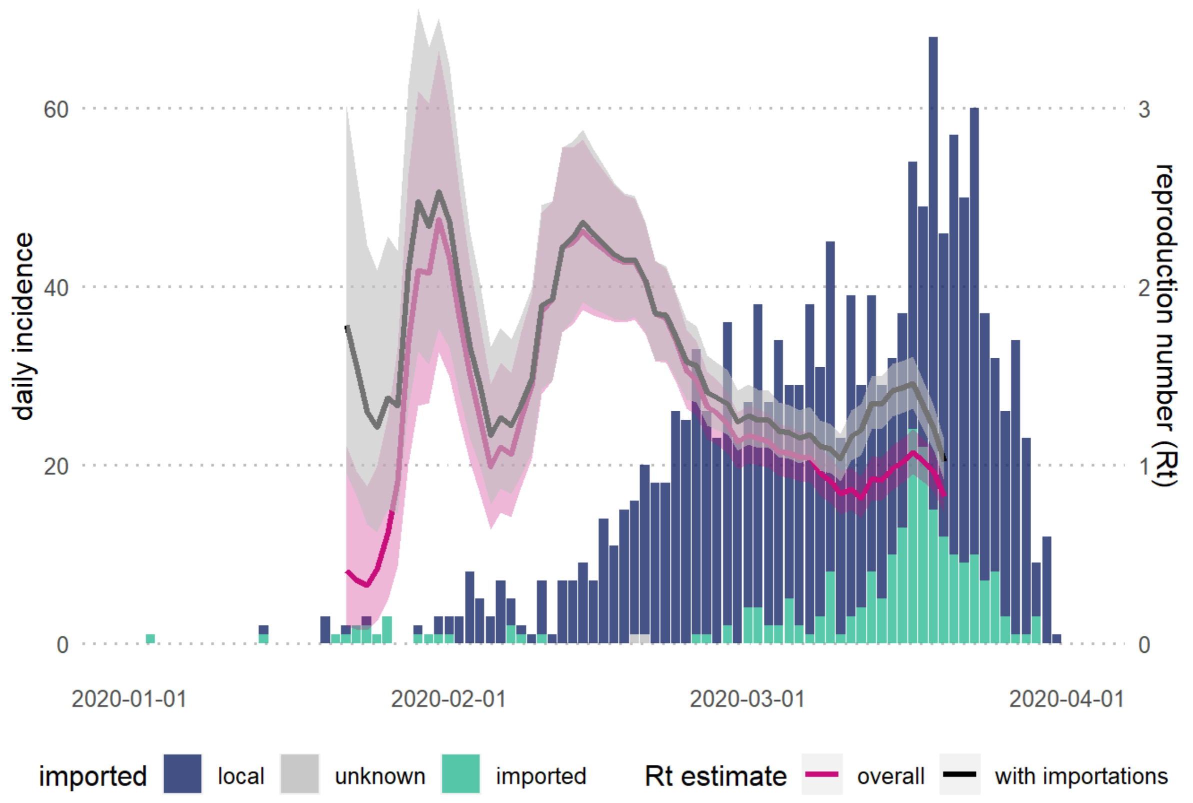

Supplementary Figure 6: Daily incidence of confirmed COVID-19 cases by date of symptom onset and the estimated time-varying reproduction number (R_t) estimated from incidence data truncated using the empirical mean onset-to-reporting delay: overall (pink) and accounting for imported cases (black). The solid line shows the median and the shaded area the 95% credible interval.

Supplementary Table 1: Summary of national and regional COVID-19 interventions up to 31 March 2020

region	Date	classification	intervention	url
**national**	2020/01/27	change in legal scope	PM designates COVID as infectious disease under infectious diseases control law	http://www.asahi.com/ajw/articles/AJ202001270041.html
**national**	2020/01/27	change in legal scope	PM designates COVID as quarantinable infectious disease under the Quarantine Act	http://www.asahi.com/ajw/articles/AJ202001270041.html
**national**	2020/01/29	repatriation	start of repatriation of Japanese citizens from Hubei province	http://japan.kantei.go.jp/98_abe/actions/202001/_00037.html
**national**	2020/01/30	expert meeting	Japan Anti-Coronavirus National Task Force established	https://japan.kantei.go.jp/98_abe/actions/202001/_00034.html
**national**	2020/02/01	travel restrictions	restrictions on foreign citizens who had visited Hubei province within 14 days	https://japan.kantei.go.jp/98_abe/actions/202002/_00001.html
**national**	2020/02/01	travel restrictions	restrictions on those with a Chinese passport issued from Hubei province	https://japan.kantei.go.jp/98_abe/actions/202002/_00001.html
**national**	2020/02/01	travel restrictions	restrictions on foreign citizens who had visited Zheijang province within 14 days	https://asia.nikkei.com/Spotlight/Coronavirus/Japan-expands-entry-restrictions-to-virus-hit-Zhejiang
**national**	2020/02/01	travel restrictions	restrictions on those with a Chinese passport issued from Zheijang province	https://asia.nikkei.com/Spotlight/Coronavirus/Japan-expands-entry-restrictions-to-virus-hit-Zhejiang
**national**	2020/02/01	emergency investment in healthcare	MOH instructed municipal and prefectural governments to establish specialised COVID-19 centres and outpatient wards	https://mainichi.jp/articles/20200203/k00/00m/040/227000c
**Kanagawa**	2020/02/05	quarantine	placed the Diamond Princess under quarantine in Yokohama	http://japan.kantei.go.jp/98_abe/actions/202002/_00006.html
**national**	2020/02/05	testing policy	begun preparations to strengthen testing capacity in all prefectures	http://japan.kantei.go.jp/98_abe/actions/202002/_00006.html
**national**	2020/02/06	travel restrictions	denied entry to the MS Westerdam from Hong Kong	http://japan.kantei.go.jp/98_abe/actions/202002/_00010.html
**national**	2020/02/12	testing policy	expanded testing criteria to all those with symptoms regardless of travel history	http://japan.kantei.go.jp/98_abe/actions/202002/_00016.html
**national**	2020/02/12	financial support	financial support for small-medium businesses affected by the outbreak	http://japan.kantei.go.jp/98_abe/actions/202002/_00019.html
**national**	2020/02/14	emergency investment in healthcare	introduced coronovirus consultation system -- this was specialised covid medical services	http://japan.kantei.go.jp/98_abe/actions/202002/_00024.html
**national**	2020/02/16	expert meeting	first Novel Coronavirus Expert Meeting	https://www.kantei.go.jp/jp/singi/novel_coronavirus/senmonkakaigi/konkyo.pdf
**national**	2020/02/17	repatriation	end of repatriation of Japanese citizens from Hubei province	http://japan.kantei.go.jp/98_abe/actions/202001/_00037.html
**national**	2020/02/17	public information campaign	released national guidelines on COVID-19 testing	https://www.mhlw.go.jp/content/10900000/000607629.pdf
**national**	2020/02/24	expert meeting	president convened ministerial meeting in response to confirmed human-to-human transmission	http://japan.kantei.go.jp/98_abe/actions/202001/_00024.html

**national**	2020/02/24	expert meeting	second Novel Coronavirus Expert Meeting	https://www.nippon.com/en/news/yjj2020022400554/japanese-experts-discuss-basic-coronavirus-policy.html
**national**	2020/02/25	combined		
**national**	2020/02/25	public information campaign	introduction of the basic policies for novel coronavirus disease control	https://www.kantei.go.jp/jp/singi/novel_coronavirus/th_siryou/kihonhousin.pdf
**national**	2020/02/25	cancel public events	request for the suspension of large-scale gatherings	https://www.mhlw.go.jp/content/10200000/000603611.pdf
**national**	2020/02/25	stay home request	asked all those with cold symptoms to stay home	https://www.nippon.com/en/news/l00269/coronavirus-basic-policy-impacts-japan%e2%80%99s-health-education-systems.html?cx_recs_click=true
**national**	2020/02/25	contact tracing	established the Cluster Response Team to identify small clusters of infections	https://www.mhlw.go.jp/stf/newpage_09743.html
**Hokkaido**	2020/02/26	school closure	closure of elementary, junior high and high schools	https://english.kyodonews.net/news/2020/02/b95d548f279f-hokkaido-eyes-temporary-shutdown-of-public-schools-to-fight-virus.html
**national**	2020/02/27	emergency investment in healthcare	expand national health insurance to cover COVID-19 testing	https://www.nippon.com/en/news/ntv20200227002/coronavirus-national-health-insurance-to-cover-virus-test.html?cx_recs_click=true
**Hokkaido**	2020/02/28	state of emergency	declaration of a new coronavirus emergency	https://www.pref.hokkaido.lg.jp/ss/tkk/koronaseng en.html
**national**	2020/03/02	school closure	closure of elementary, junior high and high schools	https://english.kyodonews.net/news/2020/02/c3c57bbce11d-breaking-news-govt-will-ask-all-schools-in-japan-to-shut-for-virus-fears-abe.html
**national**	2020/03/09	expert meeting	reconvened the expert meeting	https://www.mhlw.go.jp/content/10900000/000608425.pdf
**national**	2020/03/26	expert meeting	reconvened the expert meeting	https://www.mhlw.go.jp/content/10900000/000620826.pdf
**Tokyo**	2020/03/30	stay home request	request residents to refrain from non-essential outings for two weeks	https://japantoday.com/category/national/Koike-calls-for-fewer-outings-says-state-of-emergency-up-to-PM

Supplementary Table 2: Age-specific transmission probability (95% CI, 3 d.p) matrix for all provinces in the first wave of the epidemic in Japan (N=1276). The probability is defined as the frequency of reported transmission events from the infector age-group to the infectee age-group divided by the total number of linked transmission pairs

	**Infector**									
**Infectee**	**0-9**	**10-19**	**20-29**	**30-39**	**40-49**	**50-59**	**60-69**	**70-79**	**80-89**	**90+**
**0-9**	0<br>(0-0)	0.002<br>(0.002-0.006)	0<br>(0-0)	0.006<br>(0.002-0.013)	0.003<br>(0-0.008)	0.005<br>(0-0.011)	0<br>(0-0)	0<br>(0-0)	0<br>(0-0)	0<br>(0-0)
**10-19**	0<br>(0-0)	0<br>(0-0)	0.002<br>(0-0.005)	0.002<br>(0-0.005)	0.003<br>(0-0.008)	0.003<br>(0-0.008)	0<br>(0-0)	0.002<br>(0-0.005)	0<br>(0-0)	0<br>(0-0)
**20-29**	0<br>(0-0)	0<br>(0-0)	0.034<br>(0.021-0.049)	0.006<br>(0-0.013)	0.005<br>(0-0.011)	0.026<br>(0.013-0.039)	0.015<br>(0.006-0.024)	0.005<br>(0-0.011)	0.006<br>(0.002-0.013)	0<br>(0-0)
**30-39**	0.002<br>(0.002-0.006)	0<br>(0-0)	0.005<br>(0-0.011)	0.015<br>(0.006-0.024)	0.006<br>(0-0.013)	0.006<br>(0.002-0.013)	0.013<br>(0.005-0.023)	0<br>(0-0)	0.002<br>(0-0.005)	0<br>(0-0)
**40-49**	0<br>(0-0)	0<br>(0-0)	0.01<br>(0.003-0.018)	0.018<br>(0.008-0.029)	0.034<br>(0.021-0.05)	0.026<br>(0.015-0.041)	0.011<br>(0.003-0.021)	0.015<br>(0.006-0.026)	0.01<br>(0.003-0.018)	0<br>(0-0)
**50-59**	0<br>(0-0)	0<br>(0-0)	0.008<br>(0.002-0.015)	0.01<br>(0.003-0.018)	0.024<br>(0.013-0.036)	0.073<br>(0.055-0.094)	0.041<br>(0.026-0.058)	0.024<br>(0.015-0.037)	0.023<br>(0.011-0.036)	0.006<br>(0.002-0.013)
**60-69**	0<br>(0-0)	0<br>(0-0)	0.01<br>(0.003-0.018)	0.01<br>(0.003-0.019)	0.013<br>(0.005-0.023)	0.047<br>(0.031-0.065)	0.065<br>(0.047-0.086)	0.042<br>(0.028-0.058)	0.023<br>(0.011-0.037)	0.011<br>(0.003-0.019)
**70-79**	0<br>(0-0)	0<br>(0-0)	0<br>(0-0)	0.003<br>(0-0.008)	0.013<br>(0.005-0.023)	0.019<br>(0.01-0.031)	0.029<br>(0.016-0.042)	0.060<br>(0.042-0.078)	0.021<br>(0.011-0.032)	0.002<br>(0-0.005)
**80-89**	0<br>(0-0)	0<br>(0-0)	0<br>(0-0)	0<br>(0-0)	0.002<br>(0-0.005)	0.019<br>(0.01-0.032)	0.016<br>(0.006-0.026)	0.013<br>(0.005-0.023)	0.034<br>(0.023-0.05)	0.003<br>(0-0.008)
**90+**	0<br>(0-0)	0<br>(0-0)	0<br>(0-0)	0<br>(0-0)	0.003<br>(0-0.008)	0.013<br>(0.005-0.023)	0.011<br>(0.003-0.021)	0.005<br>(0-0.011)	0.002<br>(0-0.005)	0.003<br>(0-0.008)

The onset of pneumonia varies most significantly between youngest and oldest age groups (Supplementary Table 3). We also estimate significant differences in the onset of cough between 20-29 years and 60-69 or 80+ years and between 50-59 years and 80+ years; the onset of fatigue between 0-19 years and 20-29 or 60-69 years and between 50-59yo and 80+yo; and the onset of respiratory symptoms between 50-59 and 80+ years.

*Supplementary Table 3: Summary of p-values of the Kolmogorov-Smirnov statistic to assess whether samples of time from symptom onset to first occurrence of a specific symptom from different age groups are drawn from the same distribution. Only rows where a significant p-value appears are shown with minimum p-value per row shown in the "minimum p-value" column. Significance symbols are as follows: * <0.05, ** <0.01, *** <0.001 and significant results are highlighted in blue.*

Symptom					Ages			
**Cough**	**Fatigue**	**Fever**	**Pneumonia**	**Respiratory**	**Age group 1**	**Age group 2**	**Minimum p-value**	**Significance**
**0.46**	0.02	0.6	0.56	0.99	0-19	20-29	0.02	*
**0.8**	0.08	0.58	0.02	0.99	0-19	50-59	0.02	*
**0.96**	0.04	0.84	0.28	0.61	0-19	60-69	0.04	*
**0.87**	0.11	0.85	0.02	0.86	0-19	70-79	0.02	*
**0.25**	0.47	0.6	0	0.61	20-29	40-49	0	**
**0.76**	0.91	1	0	0.9	20-29	50-59	0	**
0.02	0.89	0.98	0.15	0.44	20-29	60-69	0.02	*
**0.38**	0.75	1	0	0.83	20-29	70-79	0	***
0	0.14	0.65	0.04	0.1	20-29	80+	0	**
**0.99**	0.12	0.99	0.05	0.27	30-39	50-59	0.05	*
**0.21**	0.74	0.87	0.03	0.06	50-59	60-69	0.03	*
0.03	0.03	0.28	0.29	0.03	50-59	80+	0.03	*
**0.47**	**0.94**	**1**	0.04	**0.75**	**60-69**	**70-79**	**0.04**	*

References

1. Ministry of Health Labour and Welfare Japan. オープンデータ｜厚生労働省. https://www.mhlw.go.jp/stf/covid-19/open-data.html. Accessed 24 Aug 2021.
